# Behind the COVID-19 Curtain: A Rare Case of Autochthonous Pneumonic Tularemia in Portugal

**DOI:** 10.7759/cureus.99693

**Published:** 2025-12-20

**Authors:** Christine Canizes Paiva, David Lopes Sousa, João Pina Cabral, João Rua

**Affiliations:** 1 Internal Medicine, Unidade Local de Saúde de Coimbra, Coimbra, PRT; 2 Intensive Care Medicine, Unidade Local de Saúde de Coimbra, Coimbra, PRT

**Keywords:** covid-19, francisella tularensis, portugal, public health, pulmonary tularemia, tularemia

## Abstract

The presence of *Francisella tularensis *has been well documented in Portugal; however, reports of human infection remain rare. We report the case of a 63-year-old woman presenting with pneumonia and SARS-CoV-2 infection. The relapsing course of infection, partial response to fluoroquinolone and macrolide antibiotics, and epidemiological background led to the suspicion of an underlying zoonotic disease. Serology later confirmed *F. tularensis* infection, and the patient improved under directed therapy. To the best of our knowledge, this is the first case of autochthonous pneumonic tularemia reported in Portugal.

## Introduction

Tularemia is a zoonosis caused by the Gram-negative, facultative intracellular bacterium *Francisella tularensis *[1-5]. Infection may occur through direct contact with infected animals, arthropod vectors, aerosol inhalation, or ingestion of contaminated food or water [1-5]. Human-to-human transmission has never been reported [1,2].* F. tularensis* is a highly infectious pathogen, with multiple animal hosts, and a potential agent for bioterrorism [2,4,6]. In Europe, *F. tularensis holarctica* is the predominant subspecies associated with human disease [1-5].

In Portugal, arthropod vectors and animal hosts of *F. tularensis *have been widely reported [1]. Epidemiologic studies have also identified an 8.9% antibody prevalence, as well as* F. tularensis holarctica* in human, lagomorph, and arthropod samples [1,7]. However, only one human case of tularaemia has been described, and presumed to be of imported origin [7]. We report the first case of autochthonous pneumonic tularaemia in Portugal.

## Case presentation

We report the case of a 63-year-old woman presenting with a 10-day history of dry cough, dyspnea, and anorexia during the first wave of COVID-19 in Portugal. Her previous medical history was relevant for myelodysplastic syndrome under erythropoietin treatment, hypertension, and dyslipidemia. She lived in a rural area and had no history of recent travel.

**Figure 1:**
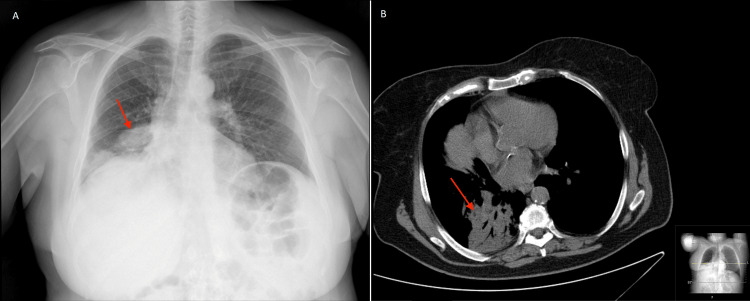
Chest X-ray (A) and CT scan (B) on admission showing lower right lobe consolidation with air bronchogram, suggestive of acute infection (red arrow).

Her physical examination was remarkable for polypnea, crackles on auscultation of the lower half of the right lung, a painful left retroauricular abrasion, and low oxygen saturation at room air. Laboratory results showed leucocytosis with neutrophilia and an elevated C-reactive protein (CRP) (Table 1). Arterial blood gas analysis confirmed type 1 respiratory failure. Chest X-ray and computed tomography (CT) revealed consolidation on the lower right lobe, consistent with acute infection (Figure 1). The nasopharyngeal swab was positive for SARS-CoV-2. The patient was admitted to the COVID-19 ward and started on standard care plus levofloxacin for suspected bacterial superinfection.

**Table 1:** Laboratory tests performed during hospital admission. CRP: C-reactive protein; Ig: immunoglobulin; PCR: polymerase chain reaction

Laboratory analysis (units)	Results (reference values)
Leucocytes (10^9^/L)	13.8 (4.0-10.0)
Neutrophils (10^9^/L)	10.82 (1.50-7.70)
CRP (mg/dL)	13.45 (<0.50)
Blood cultures	Negative
Urinary *Streptococcus* and *Legionella* spp. antigens	Negative
Bronchial secretion PCR panel: Adenovirus, coronavirus (including SARS-CoV-2), human metapneumovirus, human rhinovirus, human enterovirus, influenza A and B, parainfluenza 1-4, respiratory syncytial virus, *Bordetella pertussis*, *Chlamydophila pneumoniae*, and *Mycoplasma pneumoniae*	Negative
Aerobic culture of respiratory secretions	Negative
Anaerobic culture of respiratory secretions	Negative
*Brucella* spp. IgM/IgG	Negative
*Coxiella *burnetti IgG	Negative
*Borrelia* spp. IgM/IgG	Negative
*Legionella *spp. IgG/IgM	Negative
*Rickettsia *spp. IgM/IgG	Negative
Francisella tularensis	Positive

On day 7, in the COVID-19 ward, the patient maintained a fever and worsening cough, motivating escalation of antibiotic therapy to ceftazidime plus vancomycin, despite transitory improvement of her respiratory insufficiency. Repeated blood cultures and urinary *Streptococcus and Legionella spp*. antigens were negative (Table 1), and CRP continued to rise. Due to her worsening status, the patient was admitted to the COVID-19 Intermediate Care Unit (CICU) on day 15.

**Figure 2:**
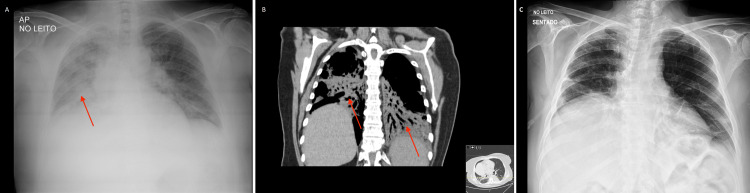
Imaging evolution during hospitalization. (A) Chest X-ray before admission to the Intensive Care Unit (red arrow). (B) Coronal cut of chest CT showing bilateral pneumonia (two red arrows). (C) Chest X-ray after five-day treatment with azithromycin, showing resolving pneumonia.

At this point, we reviewed the patient’s history and discovered that she had close contact with unvaccinated animals in her home, namely cats, dogs, chickens, and rabbits. Levofloxacin had been stopped early, and there had been no subsequent antibiotic coverage for atypical microorganisms. The patient had also developed a maculopapular rash involving the abdomen and thighs and a persistent fever higher than 39°C. Repeat chest X-ray showed worsening alveolar infiltrates on the right lung (Figure 2A).

A broad serological panel was ordered, and the patient was started on piperacillin-tazobactam, azithromycin, and vancomycin. On day 19, despite escalation of care, the patient’s condition kept worsening, and she was admitted to the Intensive Care Unit (ICU). She remained under invasive mechanical ventilation for six days. Antibiotics were stopped on the third day of ICU stay, owing to a belief that the patient’s condition had deteriorated due to COVID-19. Bronchial secretions obtained by bronchoscopy were negative for adenovirus, coronavirus (including SARS-CoV-2), human metapneumovirus, human rhinovirus, human enterovirus, influenza A and B, parainfluenza 1-4, respiratory syncytial virus*, Bordetella pertussis, Chlamydophila pneumoniae, and Mycoplasma pneumoniae *(Table 1). Repeated culture of respiratory secretions was negative for aerobic and anaerobic bacteria (Table 1). Nevertheless, the patient’s condition improved, she was successfully extubated, and returned to CICU on day 28 of her hospital stay.

On her third day of CICU readmission, the patient once again presented with worsening respiratory failure, with chest CT showing bilateral pneumonia (Figure 2B). Pending serological results, and considering that all previous tests had returned negative alongside the strong epidemiological context, we considered the patient to present with recurring atypical pneumonia of probable zoonotic etiology. Azithromycin was restarted, and after five days, the patient had clearly improved, with resolution of her fever and respiratory failure, and clearance of pulmonary infiltrates on chest X-ray (Figure 2C). Serology results then came back negative for *Brucella, Coxiella, Borrelia, Legionella*, and *Rickettsia spp.*, but positive for *F. tularensis *(Tables 1-2). Seroconversion was later confirmed at the National Reference Laboratory (Table 2). We substituted azithromycin with doxycycline, and the patient was discharged seven days later with a prescription for a total of 14-day-long treatment. We also contacted the patient's local public health team, who liaised with local veterinary offices to evaluate the patient's rabbits.

**Table 2:** Tube agglutination titers for Francisella tularensis showing IgG and IgM seroconversion with a five-fold increase. ^*1^ Acute phase sample obtained on the third week of illness. ^*2^ Convaslescent phase sample was obtained three weeks after the acute phase sample.

*Francisella tularensis *serology (tube agglutination titer)			
Acute phase sample^*1^		Convalescent phase sample^*2^	
IgG 1:160	IgM 1:320	IgG 1:640	IgM 1:1600

Follow-up at three-, six-, and 12-month post-discharge revealed no relapse of symptoms and no further need for antibiotic therapy.

## Discussion

Tularemia initially presents with acute-onset non-specific symptoms, including fever (38-40°C), chills, myalgia, and malaise [1,2,4]. Incubation period averages three to five days but may reach three weeks [1-6]. Different clinical syndromes develop according to route of infection: ulceroglandular, glandular, oculoglandular, oropharyngeal, pneumonic, typhoidal, and septic [1-6].

In ulceroglandular tularemia, a single, soft, painless ulcer manifests at the site of inoculation (from direct contact with infected animals or vector bite), accompanied by regional lymphadenopathy [1-4]. Glandular tularemia is similar, but no inoculation ulcer is discernible [1-4]. Oculoglandular tularemia is characterized by conjunctivitis, conjunctival ulcers, and localized adenopathy following direct eye contamination [1-4]. Ingestion of contaminated food or water is associated with oropharyngeal tularemia and the development of exudative pharyngitis and tonsilitis, pharyngeal ulcers, and cervical lymphadenitis [1-4]. Typhoidal tularemia presents with systemic, nonspecific symptoms and an unknown route of transmission, although intense diarrhea has been described [1,2]. Septic tularemia may develop as a complication of other disease forms and can progress to life-threatening systemic inflammation and organ dysfunction [1,2,4].

Our patient presented with pneumonic tularemia, which may occur from aerosol inhalation or from hematogenous dissemination from other disease forms [1,2]. Considering the painful left retroauricular lesion noted on admission, the latter cannot be dismissed in our patient. Typical symptoms of this form of tularemia include cough, dyspnea, pleuritic chest pain, and high fever [1,2]. Although cultures remain the gold standard [1,2], diagnosis is mostly confirmed by serology and clinical findings under the appropriate epidemiological history [1,4,5], as was the case with our patient.

Over 50% of patients require hospitalization [3]. Aminoglycosides constitute first-line treatment, but success has also been reported with tetracyclines and quinolones [2,4,6], as well as azithromycin in pregnant women [4]. We believe intermittent exposure to these antibiotics may explain the relapsing course of our patient’s disease. Furthermore, good response with azithromycin may be related to the presence of *F. tularensis* erythromycin-sensitive biovar I, common in Western Europe [5,6].

To the best of our knowledge, this is the first reported case of SARS-CoV-2 and *F. tularensis* coinfection. It is also only the second reported case of tularemia from Portugal [7], and the first one presenting with pneumonia and of autochthonous origin.

## Conclusions

Looking back, we believe that this patient presented with *F. tularensis* pneumonia on admission, although the symptoms were masked by SARS-CoV-2 coinfection. This highlights the need for frequent reassessment of these patients, especially when the clinical course is not linear, as well as the high degree of suspicion that is necessary for the diagnosis of tularemia. It also demonstrates the importance of thoroughly reviewing the clinical history in any clinical scenario and multidisciplinary discussion of complex cases to avoid observation and clinical reasoning biases.
